# Automatic identification of atypical clinical fMRI results

**DOI:** 10.1007/s00234-020-02510-z

**Published:** 2020-08-18

**Authors:** J. Martijn Jansma, Geert-Jan Rutten, Lenny E. Ramsey, T. J. Snijders, Alberto Bizzi, Katharina Rosengarth, Frank Dodoo-Schittko, Elke Hattingen, Mar Jiménez de la Peña, Gord von Campe, Margit Jehna, Nick F. Ramsey

**Affiliations:** 1 Brain Center, Department of Neurology & Neurosurgery, University Medical Center Utrecht, Utrecht, The Netherlands; 2 Department of Neurosurgery, Elisabeth-TweeSteden Hospital, Tilburg, The Netherlands; 3 Department of Neurosurgery, McGovern Medical School, University of Texas Health Science Center, Houston, TX USA; 4 Neuroradiology Unit, Istituto Clinico Humanitas IRCCS, Rozzano, Milan, Italy; 5 Institute for Experimental Psychology, University of Regensburg, Regensburg, Germany; 6 Medical Sociology, Institute for Epidemiology and Preventive Medicine, University of Regensburg, Regensburg, Germany; 7 Institute of Neuroradiology, Goethe University, Frankfurt, Germany; 8 Diagnostic Imaging Department, Hospital Universitario QuirónSalud, Madrid, Spain; 9 Department of Neurosurgery, Medical University of Graz, Graz, Austria; 10 Division of Neuroradiology, Vascular and Interventional Radiology, Medical University of Graz, Graz, Austria; 11 Braincarta BV, Utrecht, The Netherlands

**Keywords:** Functional MRI, Motor cortex, Language, Brain function, Clinical fMRI

## Abstract

**Purpose:**

Functional MRI is not routinely used for neurosurgical planning despite potential important advantages, due to difficulty of determining quality. We introduce a novel method for objective evaluation of fMRI scan quality, based on activation maps. A template matching analysis (TMA) is presented and tested on data from two clinical fMRI protocols, performed by healthy controls in seven clinical centers. Preliminary clinical utility is tested with data from low-grade glioma patients.

**Methods:**

Data were collected from 42 healthy subjects from seven centers, with standardized finger tapping (FT) and verb generation (VG) tasks. Copies of these “typical” data were deliberately analyzed incorrectly to assess feasibility of identifying them as “atypical.” Analyses of the VG task administered to 32 tumor patients assessed sensitivity of the TMA method to anatomical abnormalities.

**Results:**

TMA identified all atypical activity maps for both tasks, at the cost of incorrectly classifying 3.6 (VG)–6.5% (FT) of typical maps as atypical. For patients, the average TMA was significantly higher than atypical healthy scans, despite localized anatomical abnormalities caused by a tumor.

**Conclusion:**

This study supports feasibility of TMA for objective identification of atypical activation patterns for motor and verb generation fMRI protocols. TMA can facilitate the use and evaluation of clinical fMRI in hospital settings that have limited access to fMRI experts. In a clinical setting, this method could be applied to automatically flag fMRI scans showing atypical activation patterns for further investigation to determine whether atypicality is caused by poor scan data quality or abnormal functional topography.

## Introduction

Functional MRI (“fMRI”) is one of the most popular and widely used brain activation measurement tools in cognitive neuroscience. fMRI is a non-invasive imaging modality with a spatial resolution that is high compared with other non-invasive functional imaging methods, such as EEG. It is also relatively easily accessible due to the wide availability of MRI scanners, particularly in clinical centers. These characteristics are important reasons why the development of fMRI has had a strong impact on neuroscience.

It is also considered to carry a strong potential for clinical applications. Clinical applications of fMRI have mostly centered on presurgical use for patients with a brain tumor and patients with epilepsy. In both fields, several reviews have concluded that fMRI can provide important information for clinical care [1–5]. For instance, several studies have indicated that for language dominance, fMRI shows good agreement with invasive clinical measures such as the Wada test [6–9]. It has also been shown to be helpful in providing information about brain function topography prior to surgery for brain tumor patients [3, 10–16] and epilepsy patients [17], although some limitations have also been described [18].

One important issue limiting the use of fMRI in a clinical setting is the quality of the results which can vary considerably due to the complex process of fMRI acquisition. It is far from straightforward to determine, even for experts, whether the quality of an fMRI scan has been compromised by movement, task non-compliance, or other disruptions during acquisition. Additionally, brain activation patterns from neurological patients can deviate strongly from what is expected in location, extent, and magnitude of activated brain areas due to the neurological disorder [3, 19]. This could further complicate the problem of detecting invalid scans in a clinical setting. While certain measures indicating the quality of a scan are currently available, there is no general expert agreement on their value for accepting or rejecting a clinical fMRI activity map. The effect of some other factors, such as task compliance, can currently only be determined by expert evaluation. Thus, the determination of the quality of an fMRI scan currently requires expertise that is not readily available in clinical settings. A fast, automated, and objective first selection method for identifying atypical fMRI scans would greatly facilitate clinical use of fMRI.

**Fig. 1:**
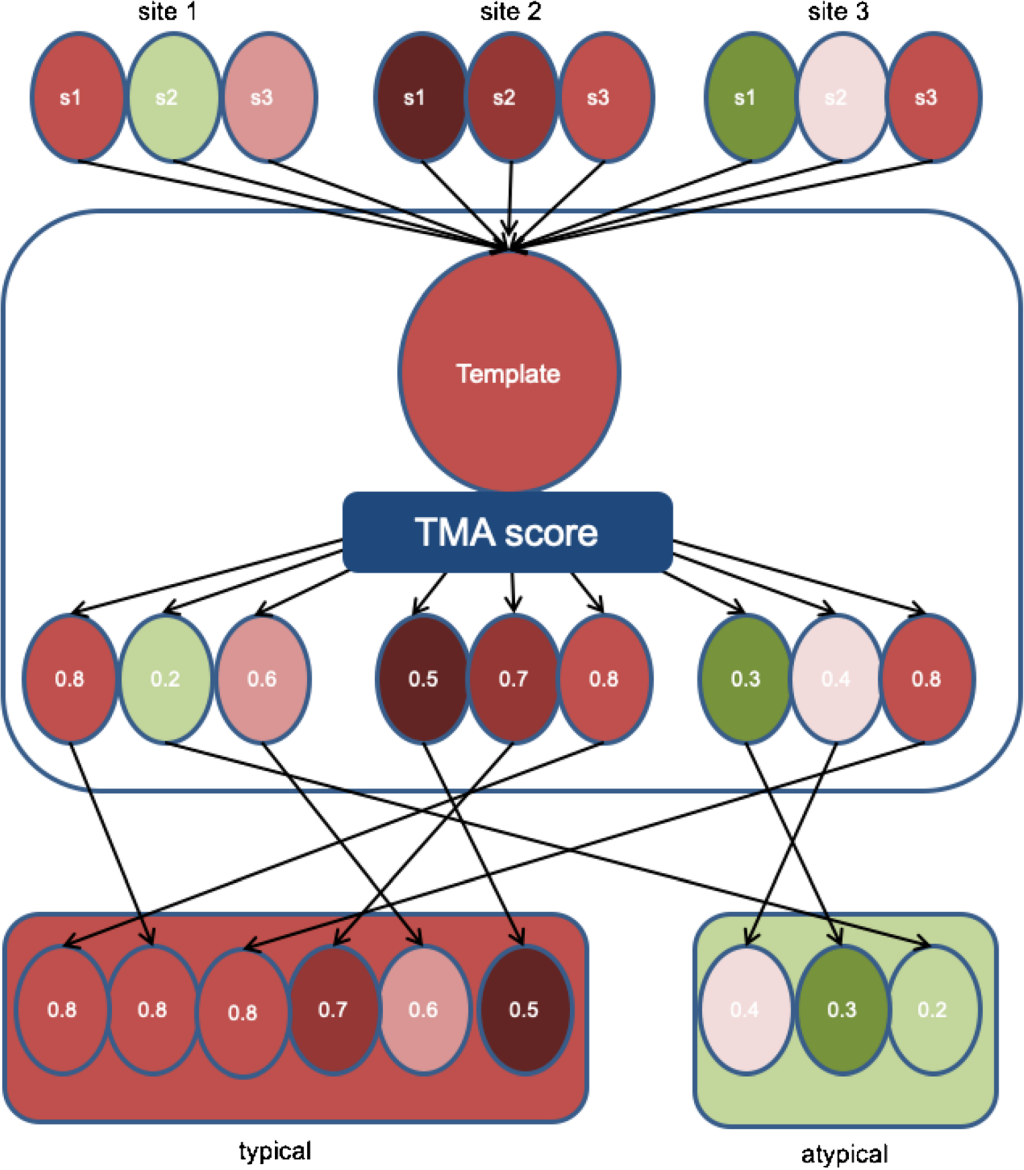
Schematic overview of the application of automatic assessment of typicality for clinical fMRI

In this report, we introduce a method that can provide a fast, automatic, and objective determination of the atypicality of an activation pattern. The approach is based on the notion that the performance of an fMRI task affects the whole brain, rather than only those parts that exceed a statistical threshold. We exploit this factor in our proposed method to determine an objective value describing the atypicality of an activation pattern. This value is based on a whole brain voxel by voxel correlation of each individual activity map with an independent template activity (“template matching analysis” or “TMA”; see Fig. 1).

In order to test the validity of the method, we analyzed fMRI from healthy controls collected at seven clinical centers in Europe with two standardized fMRI tasks to map hand motor and language function. Participating centers were all members of the European Low Grade Glioma Network (www.braintumours.eu). The tasks have previously been described for presurgical localization of motor [11, 20, 21] and language function [22, 23]. Additionally, we used this dataset to artificially create atypical activity patterns due to incorrect analysis, incorrect orientation, or incorrect task execution. This set was used to test the feasibility of our method to automatically identify scans with atypical activation patterns.

We also applied our method on a series of 32 clinical brain tumor verb generation scans from consecutive patients considered for surgery at the UMC Utrecht. The main goal of including the patient data in the current manuscript was to provide proof of principle for our presented method, specifically to test if the presence of a tumor would not affect the full brain activation patterns in such a severe manner that all patterns would become atypical and thus invalidate our approach.

## Method

### Subjects

For this study, fMRI data were included from centers in Austria (Graz), Germany (Frankfurt and Regensburg), Italy, (Milan), Spain (Madrid), and the Netherlands (Utrecht, Tilburg) (Table 1). This approach allows for assessment of differences between centers in situations that are most comparable to a real-life application of the protocols, hence with local language and routines.

**Table 1:** Scan characteristics per site

Site	Country	Brand	Model	T	Pulse sequence	TE	TR	FA	Reps
1	Netherlands	Philips	Achieva	3.0	PRESTO	41	0.75	10	560
2	Netherlands	Philips	Achieva	3.0	PRESTO	27	1.5	10	280
3	Spain	GE	Signa HDxt	3.0	EPI	35	2.5	85	168
4	Italy	Siemens	Avanto AB	1.5	EPI	50	2.5	85	168
5	Germany	Siemens	Allegra	3.0	EPI	35	2.5	80	168
6	Germany	Siemens	Allegra	3.0	EPI	35	2.0	80	211
7	Austria	Siemens	MAGNETOM Trio	3.0	EPI	35	2.5	85	168

Each site provided data from three male and three female healthy subjects aged between 18 and 30 years. All subjects were right-handed, with good eyesight (contact lenses were allowed), no history of neurological or psychiatric disease, and no use of medication other than contraceptives. All centers provided the data fully anonymized and devoid of any identifiable information. All participants gave approval for use of their anonymized data for the research. The Ethics Committee of the University Medical Center Utrecht determined that the healthy volunteer study did not require formal ethics approval, because all data were previously obtained and were fully anonymized, and all participants approved sharing of their fully anonymized data.

Additionally, clinical data from 32 consecutive patients who underwent surgery for low-grade glioma at the University Medical Center Utrecht over a period of 4 years (18 m/14 f, mean age 39 years, range 18–60 years) were also included. The patients at the University Medical Center Utrecht participated in a study that was approved by the Ethics Committee of the center, and signed informed consent, in accordance with the Declaration of Helsinki (2013). A general description of the tumor characteristics of the included patients is provided in Table 2.

**Table 2:** Patient demographic data and tumor description

Subject	Demo				Tumor location						Tumor histology	
	TMA	Sex	Age	Size	FRO	TMP	OCC	PAR	INS	NS	Grade	Characteristics
1	0.63	M	41	x		L					III	Anaplastic oligo-astrocytoma
2	0.51	F	37	x		R			R		II	Low-grade oligo-astrocytoma
3	0.50	M	63						L		II	Low-grade oligo-astrocytoma
4	0.59	F	21	x		R					II	Low-grade astrocytoma
5	0.34	F	49			L					II	Low-grade oligo-astrocytoma
6	0.48	M	42		L						IV	Glioblastoma multiforme
7	0.54	F	30		L					L	I	Pilocytic astrocytoma
8	0.50	M	31		L						II	Low-grade astrocytoma
9	0.23	F	59		L				L		III	Anaplastic oligo-astrocytoma
10	0.43	F	38		L						II	Low-grade oligodendroglioma
11	0.40	M	20					L			III	Anaplastic oligo-astrocytoma
12	0.51	F	37	x	M						III	Anaplastic astrocytoma
13	0.31	F	42	x		L			L		II	Low-grade oligodendroglioma
14	0.38	M	43	x	L				L		III	Anaplastic oligo-astrocytoma
15	0.50	M	60			L					IV	Glioblastoma multiforme
16	0.62	M	25		L				L		II	Low-grade astrocytoma
17	0.50	M	42			L			L		II	Anaplastic oligo-astrocytoma
18	0.68	M	34		R						II	Low-grade astrocytoma
19	0.65	M	26		L						I	Ganglioglioma
20	0.47	F	57			R			R		III	Anaplastic oligo-astrocytoma
21	0.43	M	37	x	R	R			R		III	Anaplastic astrocytoma
22	0.07	M	18					L			I	Pilocytic astrocytoma
23	0.50	M	34		R				R		III	Anaplastic oligo-astrocytoma
24	0.34	F	38			L		L			I	Unknown
25	0.67	F	33			L					I	Ganglioglioma
26	0.33	F	46			L		L			II	Low-grade astrocytoma
27	0.46	M	40			L			L		I	Pilocytic astrocytoma
28	0.46	F	40		L						IV	Glioblastoma multiforme
29	0.34	F	38	x	L						II	Low-grade astrocytoma
30	0.56	M	41	x	R	R			R		II	Low-grade astrocytoma
31	0.44	M	24	x	L						II	Low-grade oligo-astrocytoma
32	0.29	M	51	x	L						IV	Glioblastoma multiforme

*Demo* demographical data: *F* female, *M* male; size: *x* clear anatomical displacement; tumor location: *FRO* frontal lobe, *OCC* occipital, *TMP* temporal lobe, *PAR* parietal lobe, *INS* insula, *NS* not specified, *L* left hemisphere, *R* right hemisphere, *M* midline

### Task protocols

The instructions to technical personnel were written in detail in a manual describing the fMRI procedure and data storage. The tasks were explained to the volunteers in accordance with written instructions for the technicians. Volunteers did not practice the tasks before the fMRI scans. The two protocols were distributed on a DVD to be played on a DVD player and presented to subjects in the scanner. The menus and instructions on the DVD and the list of nouns for the verb generation (VG) were translated to the local language for each site. All tasks started with 15 s of rest, followed by 7 tasks blocks of 30 s, interleaved with rest periods of 30 s, and ended with a 15-s rest period. Hence, the total time for each task was equal (7 min). The following instructions were provided to each site for each protocol:

1. Finger tapping (“FT”). Instructions to subject: “You will see a circle on the screen. Sometimes it is red and sometimes it is green. When it is red you will lie still and relax. When it is green the circle will flash on and off. You will then touch each of your fingers of your right hand with the right thumb, one by one, at the rhythm of the green flashing circle. Do not move your arm. Your left hand stays relaxed for the whole task.” Frequency of the movement was 1 Hz.
2. Verb generation (“VG”). Instructions to subject: “You will see words, or black bars on the screen. When the bars appear you do nothing and lie still and relaxed. When a word appears it will be a noun. Think of what you can do with it and then imagine saying: ‘With that I can …’or ‘That I can …’ For example: when you see the word ‘chair,’ imagine saying ‘That I can sit on.’ Do not speak because then you will move your head and the scans become unusable. If you cannot think of what you can do with a word, skip it and continue with the next word.” Time per word was 3 s.

### fMRI data acquisition

For each site, only the following scan parameters were standardized: FOV 256, scan matrix 64 by 64, slice thickness (including gap) 4 mm, 30 slices in transverse orientation parallel to the Sylvian fissure, one slice above the top of the brain. All centers used a quadrature head coil. Scanner equipment and scan protocol are detailed in Table 1. Field strength varied between 1.5 and 3.0 T. Scan time per volume varied between 0.75 and 2.5 s. Five centers used an EPI sequence; two centers used a PRESTO sequence [24].

### fMRI analysis

All the fMRI datasets were sent to one site (UMC Utrecht, the Netherlands) for analysis after anonymization. All data processing and analysis was performed using IDL 8.2 (ITT Exelis Inc. McLean, VA), unless otherwise specified. All statistical tests were performed using SPSS 20.

Scans were registered to the last functional scan to correct for movement [25]. Scans were smoothed with a 3D Gaussian filter (full width at half maximum 12 mm) to minimize effects of functional anatomical differences between subjects. Scans were spatially normalized to a standard EPI template in MNI space (from the SPM5 template library) using linear transformation incorporated in FSL software [26]. All time series were normalized to a mean value of 100 per voxel to allow for comparisons between subjects. Temporal filtering was applied to remove low-frequency drifts (linear, first- and second-order slow trends [27]). All tasks were analyzed using a GLM, with the regressor of interest constructed using a canonical HRF model incorporated in SPM 5 (Wellcome Trust Center for Neuroimaging, London, UK), convolved with the boxcar input function of each protocol. For TMA, the b-maps generated by the GLM were used.

The TMA value that we introduce in this study is identical to the Pearson’s *r* value, calculated over the beta values from all voxels in a template activation pattern and an individual activation map, without application of any selection or any application of an activation threshold value. The TMA was calculated for each subject using a template based on data from the other six centers. Thus, each value was based on a comparison with an independent template (“leave-one-center-out” approach).

To examine the feasibility of TMA to automatically identify atypical activation patterns, we artificially created a large set of “atypical” scans (from the set of 42 scans) that included some common problems that can occur in a clinical setting: a first set of atypical activation patterns was created by reversing the orientation over the *y*-axis to create an incorrect left-right orientation (“REVERSED”). A second set was created by using the activation pattern from the other task (“TASK”), and a third set was constructed by applying an incorrect analysis, using task regressors that were shifted 15 s (“SHIFT”). We categorized all fMRI scans from the healthy subjects as “typical.”

A brain tumor can affect activation patterns both physically by the tumor itself and indirectly due to for instance functional plasticity. If the majority of fMRI scans of brain tumor patients show highly atypical activation patterns, the usefulness of a TMA is strongly reduced. Thus, it is important to provide a proof of principle for the feasibility of TMA for scans of patients with a brain tumor. For this reason, we applied TMA on fMRI data of 32 tumor patients at the UMC Utrecht who had performed the VG task for clinical purposes and compared the values to those of the healthy controls. The clinical fMRI procedure involves the same VG task as used in the healthy volunteers and includes training before entering the scanner. For additional validation, we compared the patient results to atypical SHIFT scans, atypical TASK scans, as well as atypical REVERSED scans, using an independent samples *t* test after a Fisher *Z* transformation was applied to each TMA value to ensure normal distribution.

### Automatic detection of atypical activation patterns

In the TMA approach, it is important to minimize incorrect identification of activity patterns, i.e., false negatives (when an atypical pattern is mistaken for being typical) and false positives (when a typical pattern is mistaken for being atypical). We examined for each task the false positive rate, after determining the lowest TMA value threshold that correctly identifies all atypical scans.

## Results

### Comparison of centers

Data from each site were compared with data from all other centers. For each site test (per task), a template was built of all data from the other six centers. For each subject in the tested site, the task b-map was compared with the template by means of the TMA calculation. Hence, from each single individual, two TMA values were obtained. These values were averaged per site and compared across centers.

**Fig. 2:**
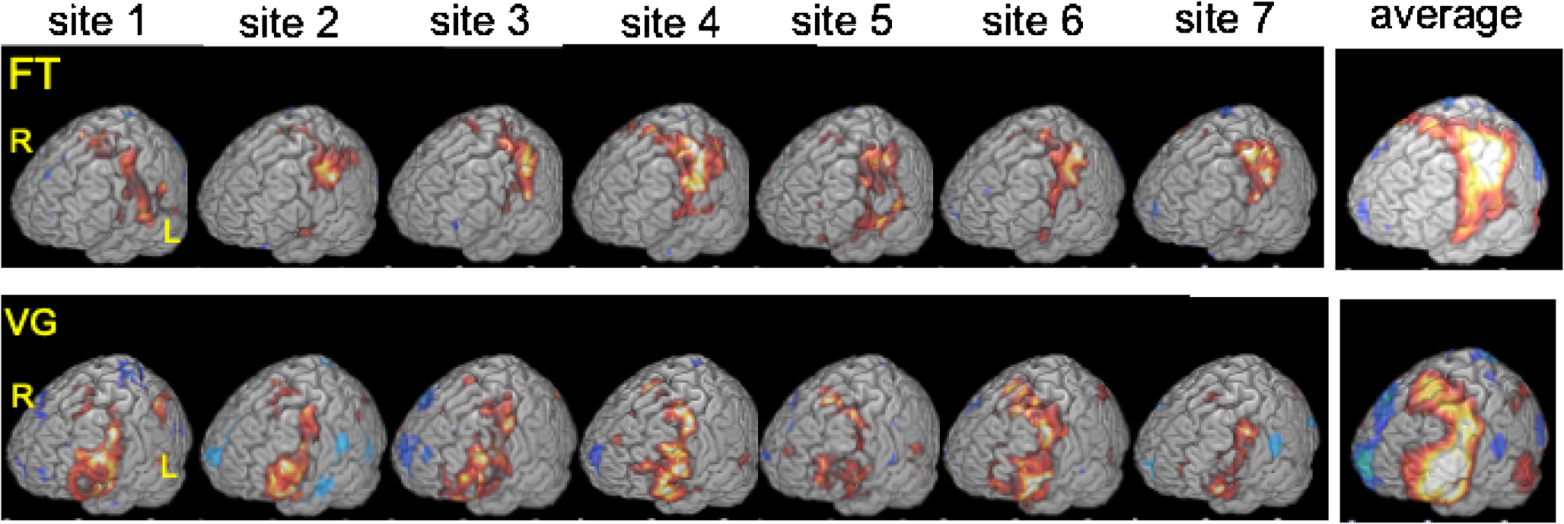
Rendered group activation patterns per site and per task protocol (|*t*| < 3.14; df = 6; positive activity in red; negative activity in blue; FT, finger tap protocol; VG, verb generation protocol; L, left; R, right)

**Fig. 3:**
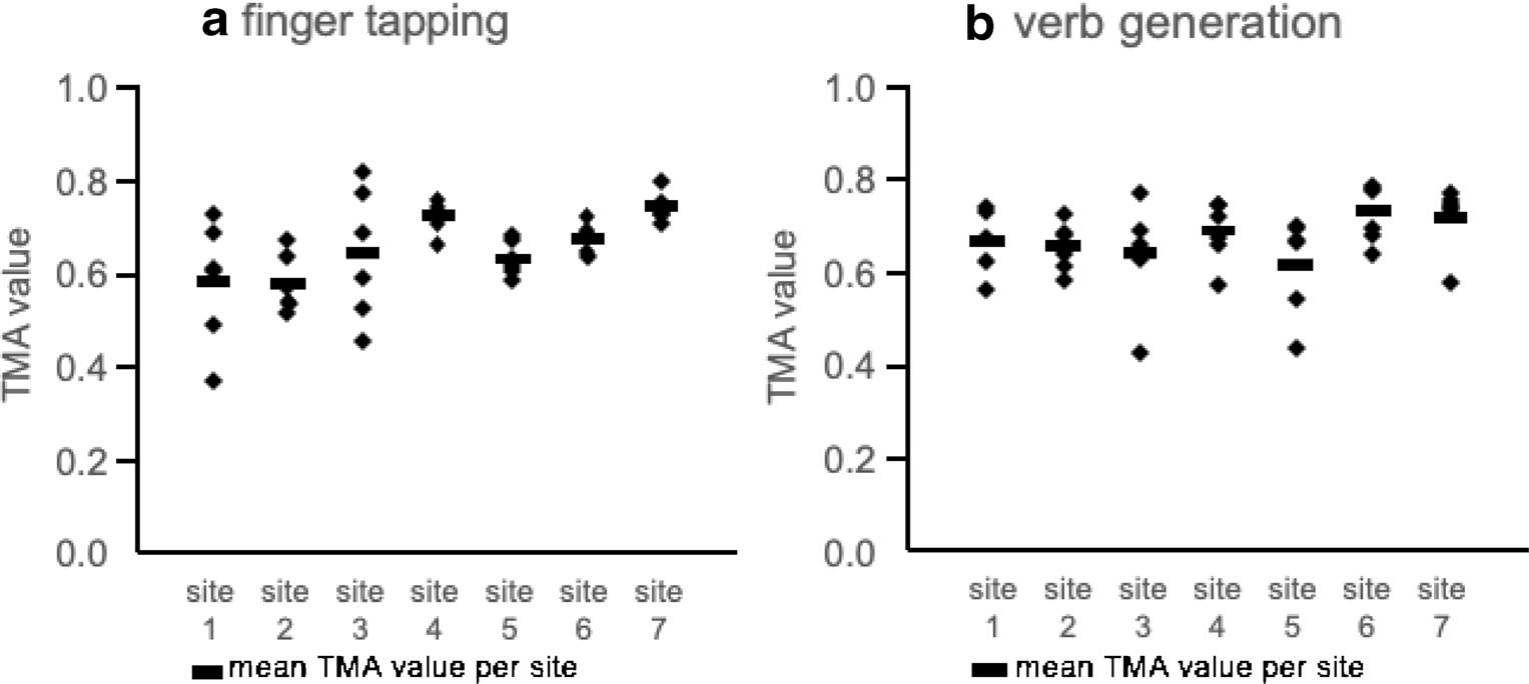
TMA results for each subject per site (**a** FT (finger tapping), **b** VG (verb generation)). Horizontal bars represent mean TMA per site. While all centers showed high TMA values, we did find a significant difference between centers in the mean values (*F*(6,35) = 3.17; *p* = 0.009), which is likely the result of slight differences in signal to noise between the scanners of the different centers

Figure 2 presents group activation maps of each site and task, as well as the grand average activation pattern over all 42 subjects. One can appreciate the likeness of activity patterns across centers. The average TMA values for each site and task are displayed in Fig. 3. Results indicate that all subjects from every site correlated high with the templates. This indicates that if a template is made based on a number of centers, the likelihood of that template being valid for evaluation of data from a new site is high.

All centers yielded a high mean TMA value for both tasks, ranging from 0.58 to 0.74 for the FT protocol and 0.66 to 0.72 for the VG protocol. TMA for individuals ranged from 0.36 to 0.82 for the FT protocol and from 0.42 to 0.78 for the VG protocol. We did find neither a difference in TMA values between the two protocols (*F*(6,35) = 1.98; *p* = 0.22) nor a site by protocol interaction (*F*(6,35) = 1.33; *p* = 0.28). Although all centers showed high TMA values, we did find a significant difference between centers in the mean values (*F*(6,35) = 3.17; *p* = 0.009).

### Classification based on TMA

**Fig. 4:**
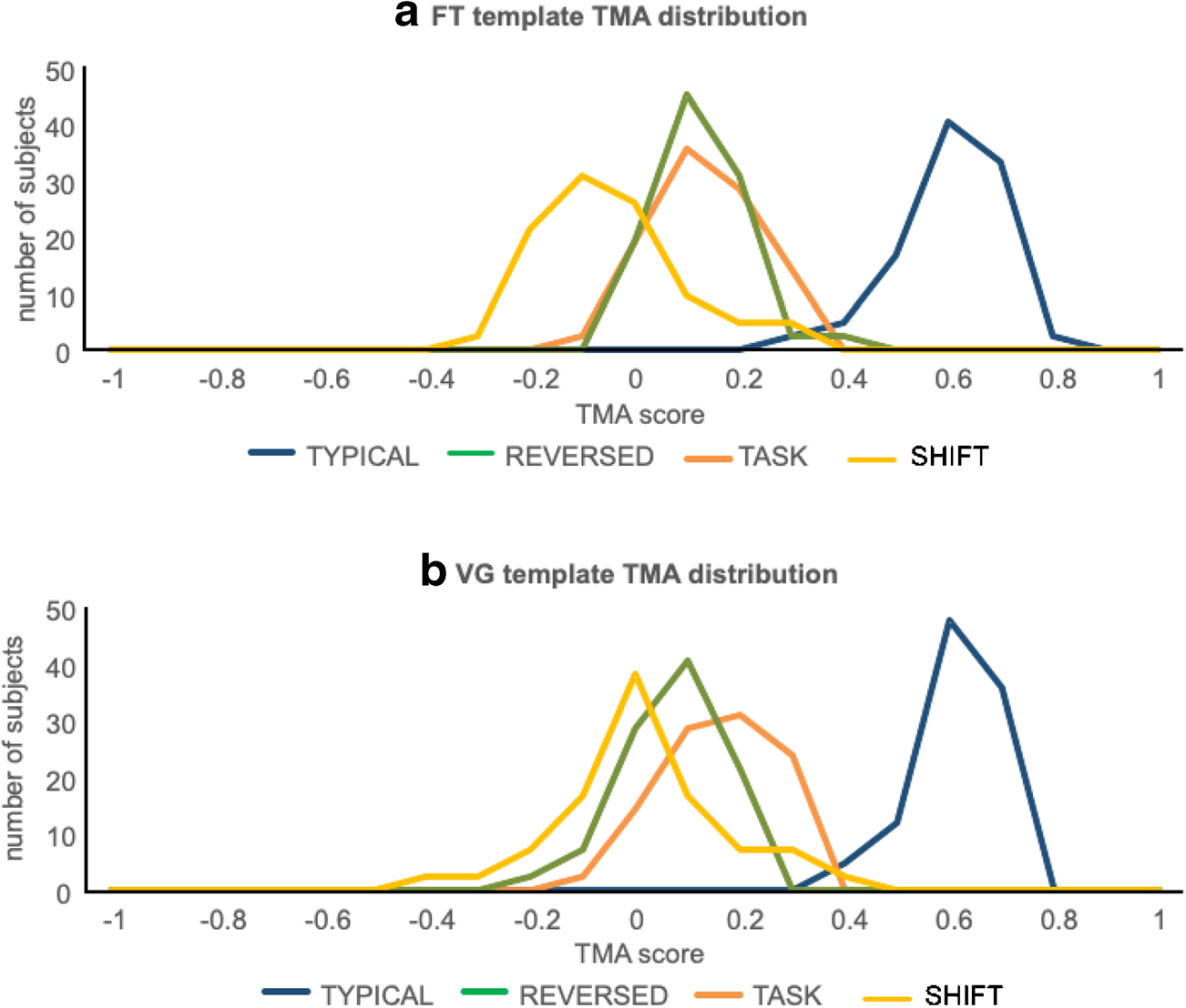
Distribution of template matching analysis (TMA) values for typical scans (“TYPICAL,” blue), as well as for atypical scans due to reversed orientation (“REVERSED,” green), incorrect task protocol (“TASK,” orange), or incorrect analysis with a time-shifted regressor (“SHIFT,” yellow). **a** Finger tapping (FT) template. **b** Verb generation (VG) template

**Fig. 5:**
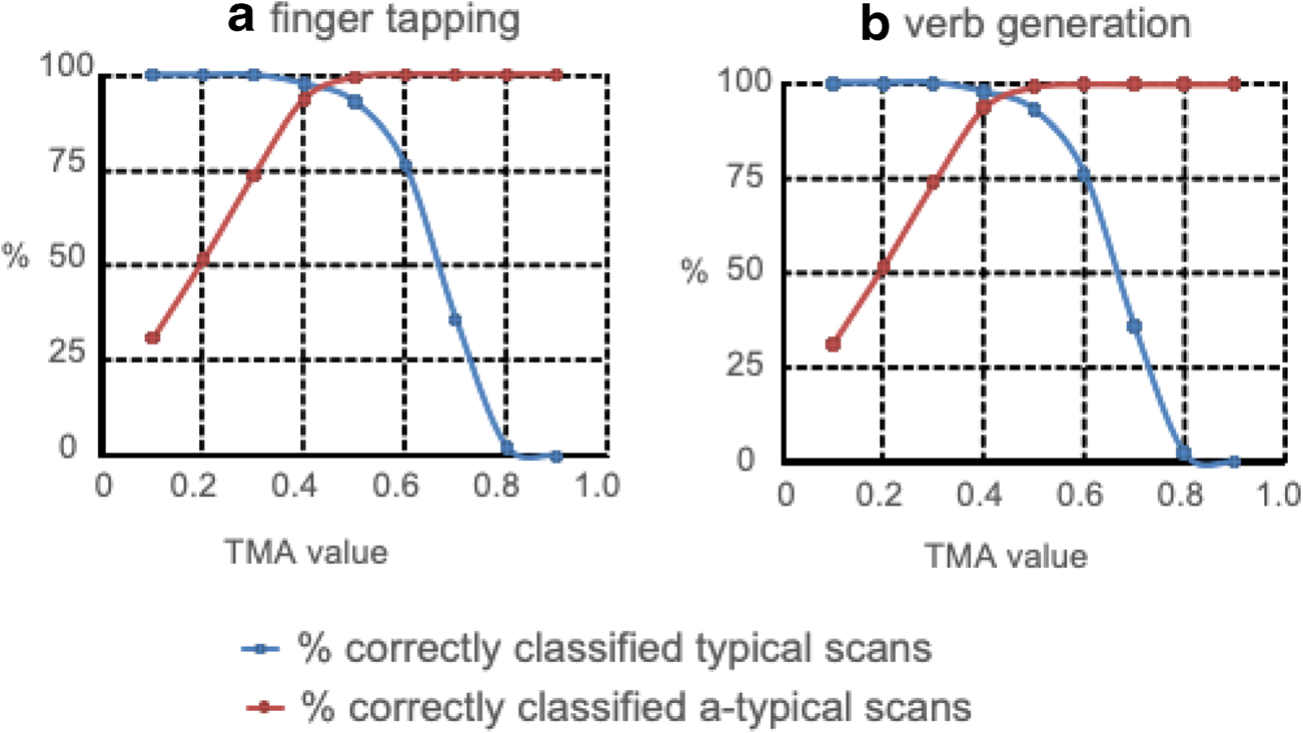
Receiver operating characteristic curve for healthy subjects data of **a** FT and **b** VG tasks. Figures display the percent correct classification (*y*-axis), for a range of TMA values

**Fig. 6:**
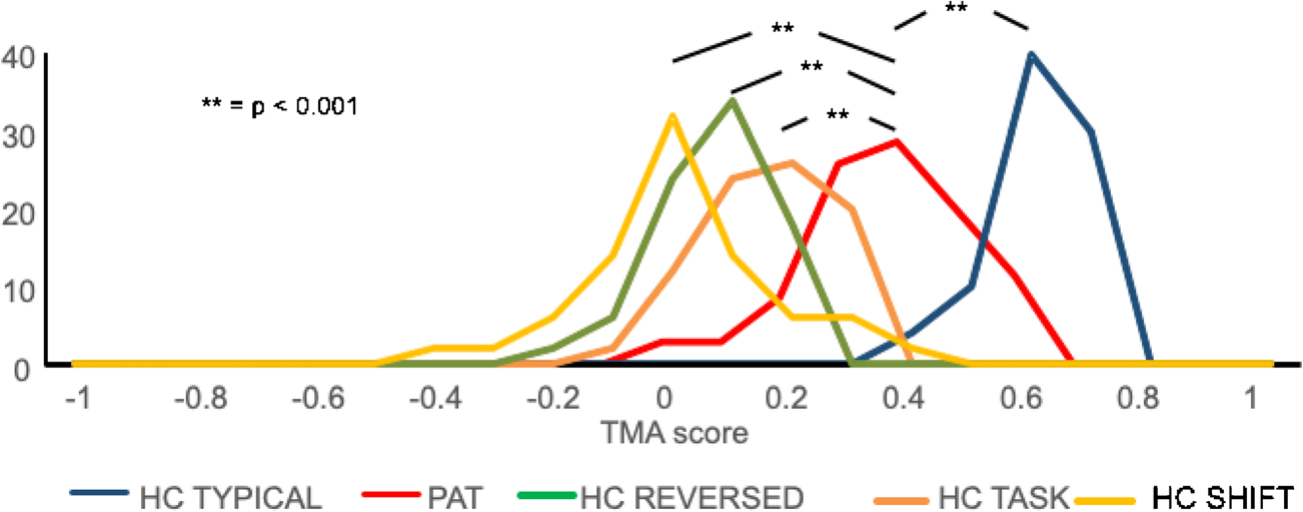
Distribution of template matching analysis (TMA) values for the verb generation (VG) template for correctly analyzed healthy controls (“TYPICAL,” blue), tumor patient data (“PAT,” red), as well as for atypical scans due to reversed orientation (“REVERSED,” green), incorrect task protocol (“TASK,” orange), or incorrect analysis with a time-shifted regressor (“SHIFT,” yellow). Patient TMA values were significantly higher than SHIFT (*t*(72) = 8.33; *p* < 0.001), TASK ((*t*(72) = 8.33; *p* < 0.001), as well as REVERSED scans ((*t*(72) = 8.33; *p* < 0.001)

To assess feasibility of using TMA for assessment of pattern typicality, we compared TMA values of all individual subjects with TMA values computed from deliberately corrupted versions of the original scans. In Fig. 4, TMA values are displayed for all typical scans, as well as for atypical scans due to incorrect orientation or incorrect task protocol. In Fig. 6, we provide “receiver operating characteristic curves” (“ROC curves”), showing the percentage correctly identified typical and atypical scans at a range of TMA threshold values for the FT and VG protocols. These figures clearly illustrate the power of the TMA method to correctly detect both typical and atypical scans for the FT and VG templates in healthy controls, as well for the VG template in patients (Fig. 5).

For the FT protocol, the average TMA value for a typical scan (correct task, correct analysis, correct orientation) was 0.65 (range 0.36–0.82). For atypical scans due to incorrect task protocol, the average TMA value was 0.24 (range − 0.07 to 0.49). Of the typical scans, 95.2% had a higher TMA value than the highest value for an incorrect task protocol, so all atypical patterns could be detected, with a percentage of missed typical scans (i.e., typical scans misidentified as atypical or “false positives”) of 4.8% (or 2 in 42).

For incorrect orientation, the average TMA value was 0.27 (range − 0.01 to 0.54). Of the typical scans, 85.7% had a TMA value higher than the highest value for a scan with incorrect orientation, so the percentage of false positives was 14.3% (6 in 42).

For incorrect analysis due to a shifted regressor, the average TMA value was 0.00 (range − 0.23 to 0.37). Of the typical scans, 97.6% had a TMA value higher than the highest value for a scan with incorrect analysis, resulting in a percentage of false positives of 2.4% (1 in 42).

Taken together, for the FT protocol, we were able to correctly classify 93.5% of the scans as typical, while detecting 100% of the atypical scans, using a TMA threshold of 0.49.

For the VG protocol, the average TMA value for a typical scan (correct task, correct analysis, correct orientation) was 0.67 (range 0.42–0.78).

For atypical scans due to an incorrect task protocol, the average TMA value was 0.25 (range − 0.10 to 0.47). Of the typical scans, 95.2% had a higher TMA value than the highest value for an incorrect task protocol, so the percentage of false positives was 4.8% (2 in 42).

For incorrect orientation, the average TMA value was 0.26 (range 0.06–0.51). Of the typical scans, 95.2% had a TMA value higher than the highest value for a scan with incorrect orientation, so the percentage of false positives was 4.8% (2 in 42).

For incorrect analysis due to a shifted regressor, the average TMA value was 0.06 (range − 0.25 to 0.43). Of the typical scans, 95.2% had a TMA value higher than the highest value for a scan with incorrect analysis, resulting in a percentage of false positives of 2.4% (1 in 42).

Taken together, for the VG protocol, we were able to correctly classify 96.4% of the scans as typical, while detecting 100% of the atypical scans, using a TMA value of 0.51.

In Fig. 6, we provide receiver operating characteristic curves (ROC curves), showing the percentage of correctly identified typical and atypical scans at a range of TMA threshold values for the FT and VG protocols. Figures 4 and 6 illustrate the ability of the TMA method to correctly distinguish between typical and atypical scans for the FT and VG templates in healthy controls.

### Correlation between TMA and strength of activation

We also evaluated if the quality of the scan, approximated by the maximum *t* value of the activation pattern, was a factor that affected the TMA value. For this reason, we calculated, for both the VG and FT protocols, the Pearson correlation between the maximum *t* value and the TMA value. The maximum *t* value did not correlate with the TMA value for either protocol (FT: *r* = 0.09; *p* = 0.58; VG: *r* = 0.14; *p* = 0.38).

### Template matching results for brain tumor patient data

We gathered 32 datasets of consecutive tumor patients who had executed the VG protocol at the UMC Utrecht for clinical purposes. Figure 5 shows the distribution of the TMA values of all patients, compared with typical healthy subject scans and the atypical sets of healthy subject scans. TMA values of the patient scans were significantly lower than those of healthy subjects (*t*(72) = 8.64; *p* < 0.001), but significantly higher than those of atypical SHIFT scans (*t*(72) = 10.72; *p* < 0.001), atypical TASK scans ((*t*(72) = 6.91; *p* < 0.001), and atypical REVERSED scans ((*t*(72) = 7.11; *p* < 0.001).

## Discussion

The main goal of this study was to evaluate a novel method for fast, objective, and automatic detection of atypical fMRI scans based on the activation patterns. The method is based on a template matching analysis (TMA). Activation patterns were collected from seven European clinical centers and two commonly used clinical fMRI tasks for localization of motor and language, and from patients with a brain tumor from the UMC Utrecht.

Results indicated that fast and automatic detection of atypical activation patterns appears to be a reachable goal in healthy subjects. For localization of motor activity, a detection rate of 93.5% of compromised scans could be achieved using the TMA approach, while for location of language activation, the VG protocol, a detection rate of 96.4% was achieved. The analysis of patient VG data suggests the feasibility of TMA in a clinical setting. Patients with a brain tumor did show slightly lower template matching values on average, which is likely due to the inclusion of poor-quality data due to movement and cognitive impairments, and anatomical and functional abnormalities related to the tumor. None of these confounders was considered for the purpose of this study, in order to obtain a conservative indication of the robustness of the TMA method.

Automatic detection of atypical activation patterns may prove helpful in clinical fMRI settings. While scans with a high TMA value can be forwarded for clinical use without need for expert quality assessment, experts can be consulted in cases where the scan has a low TMA value in order to determine the cause, such as movement, failure to comply with task rules by the patient or suboptimal analysis, versus abnormal activity due to severe anatomical deformation or functional plasticity. Thus, TMA may improve the quality of care by providing an objective and reproducible assessment of the fMRI activation pattern, and it can increase the cost efficiency by reducing the need for fMRI experts to analyze and interpret each fMRI dataset.

An alternative use of our method can be found in applications for large databases: TMA can automatically, at low cost and minimal manpower, identify datasets that are potentially compromised, thereby rapidly indicating potential site-specific problems and improving the quality of the database. This could facilitate studies that aim at gathering large databases over longer time or studies that need to pool fMRI data from various centers with potential systematic differences in quality.

It can be expected that the presence of a brain tumor changes the anatomy of a brain due to its effects on tissue integrity and displacement, as well as the functional anatomy, as it may disrupt functional networks. While the patients did display lower TMA values than healthy controls, the values were significantly higher than those of the atypical healthy subject scans (Fig. 5). A lower mean TMA value is to be expected since the set is likely to include poor-quality data (due to for instance movement, non-compliance, compromised cognitive function) as well as functional and large anatomical changes caused by the tumor.

One possible explanation for the limited tumor effects on TMA is that the protocols that were used evoked a pattern of activations and deactivations that are widespread over the brain. Thus, although the anatomy as well as functional activity pattern may change in the vicinity of the tumor, the activity pattern across the whole scan volume appears to be more stable and not severely affected by the tumor, resulting in only a somewhat lower than normal value for atypicality for most patients. Of note, task compliance and movement were not taken into account in order to obtain a conservative indication of robustness of TMA.

An efficient and reliable language protocol is a particularly important tool for presurgical planning as well as research [17, 28–31]. Clinical fMRI can locate the presence of crucial language activation in the immediate vicinity of a tumor. This would mandate a cautionary surgical approach and can be a reason to perform awake surgery or even advise against resective surgery. Importantly, fMRI may fail at the edge or within the tumor, due to abnormal vascular properties and brain tissue which can reduce the fMRI signal [32], and as such can only be used in conjunction with other clinical diagnostic modalities.

One of the important and possibly counterintuitive findings of this study is that the language protocol performed comparable with the motor protocol and showed robust results, despite the more complex nature of language production compared with motor performance and execution in four languages. This result suggests that the pattern of activity evoked by language production is as consistent over subjects as that of motor production.

Our study also demonstrated that fMRI patterns do not differ strongly between centers. Previous multisite studies, applying simple motor protocols [33–38], visual protocols [39, 40], and sometimes cognitive protocols [41–43], have consistently reported promising results for the possibility to combine fMRI data across centers. Site variation typically appears to be much smaller than subject variation (see for instance Costafreda [44] for a review). However, previous multisite studies have predominantly evaluated location or strength of activation at a specific predefined area that was expected to be activated by the protocol. TMA indicates that whole-brain activation patterns associated with language as well as motor function are consistent over centers with a considerable range in hardware and scan protocols (Table 1).

The fMRI tasks were chosen based on extensive use across centers and validation against direct electrical stimulation in brain surgery [45, 46] and electrocortical mapping [47, 48]. The tasks are easy to understand and perform for most surgery patients and yield robust levels of activation. Apart from simplicity to promote patient compliance, characteristics that may be important in robustness include a block design (as opposed to a less sensitive event-related design [49]) and non-ambiguity of the task to avoid use of different cognitive strategies. Yet, patients should be tested before an fMRI scan to ascertain cognitive capability and ability to sustain attention.

The robustness of a standardized protocol can be applied to improve reliability of clinical fMRI, facilitate analysis, and interpretation of results. The quality of a fMRI scan is vulnerable and can be affected by many factors, such as subject movement, task compliance, and incorrect analysis steps. In many cases, it is difficult to objectively judge the quality of an fMRI scan, other than through visual inspection of the activity pattern, and as of yet, there is no commonly accepted metric for quality. The current results indicate that TMA provides a simple but effective metric that can be evaluated in future studies against expert quality assessment.

Robust protocols can also facilitate studies that are plagued by low power because of difficulties in recruitment due to for example low incidence rates of specific pathologies (e.g., low-grade glioma at a particular location) or surgical procedures [50]. It allows for the possibility to pool data from various centers for clinical fMRI studies, without substantial loss in statistical power. This approach can for instance be used for patient follow-up studies that aim at examining the association between surgery and patient outcome, especially in terms of the presence of functional plasticity, or for cohort studies, where large populations are required.

There are several limitations that have to be considered in interpreting the results of this study. Importantly, while the patient results do successfully indicate that presence of a tumor does not necessarily have an invalidating impact on TMA, it will take more patient data and further expert evaluation in order to determine a firm TMA threshold for clinical use.

Our study did not evaluate the relevance of the information acquired by the task protocols but was limited to the reproducibility of the pattern across individuals. Our study did not evaluate differences in the level of the activity between centers, but instead focused on agreement between patterns. Possibly, there may have been differences in levels of activity between centers due to hardware and scan sequence differences. However, for clinical use, we do believe that the pattern of activity may be more informative than absolute levels.

Another limitation is that patients were not evaluated for quality of the scans, leaving the question about an appropriate TMA threshold for clinical use open. In the patient dataset used here, it is not known how many were of poor quality or how many exhibited lack of compliance due to cognitive or attentional deficits. This requires an extensive study comparing TMA in patient data with multiple quality measures including subjective evaluations by experts which did not fall within the scope of this proof-of-principle study. Moreover, we did not apply correction for abnormalities in anatomical structure caused by the tumor. But based on the current results, techniques that are able to correct for those, for instance, by non-linear “warping” techniques for normalizing a patient anatomical scan to a standard brain [51], are likely to further improve robustness of TMA. Finally, future studies as well as applications need to indicate if TMA can also be used to detect more subtle forms of atypical activation, for instance, if they are associated with poor task performance due to cognitive deficits.

As a final note, one could argue that head motion could increase the TMA value by generating false activations. We argue that motion artifacts can only elevate TMA if motion causes increases and decreases in various regions in such a way that they match the whole-brain template. Given the nature of data acquisition, this is highly unlikely since motion affects whole slices (and generally multiple) and is most often manifested along intensity edges (typically gray matter vs CSF) across the brain. Neither is likely to generate the template pattern. In effect, even though task-correlated motion may generate spurious significant activity along intensity edges, motion will reduce the TMA that is computed across the brain because it adds the image noise resulting from motion to the data.

In conclusion, we present a straightforward method for assessing typicality of fMRI activation patterns and validate it with correctly and incorrectly analyzed data from 42 healthy subjects from 7 centers. The results indicate that TMA is robust and detects the atypical activity patterns derived from the incorrectly analyzed quite well. The application of TMA to a series of patient data suggests that the presence of localized anatomic abnormalities does not invalidate the method (although TMA values were lower than that of healthy subjects). Of note, it is of importance for clinical fMRI to optimize the scan procedure in order to avoid artifacts, preferably by applying some sort of quality assurance.
